# A Distinctive Metabolomics Pattern Associated with the Administration of Combined Sacubitril/Valsartan to Healthy Subjects: A Kinetic Approach

**DOI:** 10.3390/ph18091264

**Published:** 2025-08-25

**Authors:** Randh AlAhmari, Hana M. A. Fakhoury, Reem AlMalki, Hatouf H. Sukkarieh, Lina Dahabiyeh, Tawfiq Arafat, Anas M. Abdel Rahman

**Affiliations:** 1 Department of Biochemistry and Molecular Medicine, College of Medicine, Alfaisal University, Riyadh 12846, Saudi Arabia; ralhmari@alfaisal.edu (R.A.); hhajeer@alfaisal.edu (H.M.A.F.); 2 Metabolomics Section, Precision Medicine Laboratory Department, Genomics Medicine Center of Excellence, King Faisal Specialist Hospital and Research Centre (KFSHRC), Riyadh 12846, Saudi Arabia; ralmalki@kfshrc.edu.sa; 3 Department of Pharmacology, College of Medicine, Alfaisal University, Riyadh 12846, Saudi Arabia; hsukkarieh@alfaisal.edu; 4 Department of Pharmaceutical Sciences, School of Pharmacy, The University of Jordan, Amman 11118, Jordan; l.dahabiyeh@ju.edu.jo; 5 Jordan Center for Pharmaceutical Research, Amman 11118, Jordan; tawfiqarafat@yahoo.com

**Keywords:** Sacubitril/Valsartan, metabolomics, LC-QToF HRMS, pyrimidine metabolism, pharmacokinetics, uridine triphosphate, heart failure

## Abstract

**Background/Objective:** Sacubitril/Valsartan are a combination drug approved for heart failure treatment, known to enhance natriuretic peptide activity and inhibit the renin–angiotensin–aldosterone system (RAAS). While its clinical efficacy is well-established, its broader impact on human metabolism remains insufficiently characterized. This study aimed to explore the time-resolved metabolic changes induced by Sacubitril/Valsartan in healthy individuals using an untargeted metabolomics approach. **Methods:** Fourteen healthy male volunteers received a single oral dose of Sacubitril/Valsartan (200 mg; 97.2 mg Sacubitril and 102.8 mg Valsartan) across two phases separated by a two-week washout period. Plasma samples were collected at eight individualized time points based on pharmacokinetic profiles. Metabolites were extracted and analyzed using high-resolution liquid chromatography–mass spectrometry (LC-QToF HRMS). Data processing included peak alignment, annotation via HMDB and METLIN, and statistical modeling through multivariate (PLS-DA, OPLS-DA) and univariate (ANOVA with FDR correction) analyses. **Results:** Out of 20,472 detected features, 13,840 were retained after quality filtering. A total of 315 metabolites were significantly dysregulated (FDR *p* < 0.05), of which 31 were confidently annotated as endogenous human metabolites. Among these, key changes were observed in the pyrimidine metabolism pathway, particularly elevated levels of uridine triphosphate (UTP) associated with cellular proliferation and metabolic remodeling. OPLS-DA models demonstrated clear separation between pre-dose and Cmax samples (R^2^Y = 0.993, Q^2^ = 0.768), supporting the robustness of the time-dependent effects. **Conclusions**: This is the first study to characterize the dynamic metabolomic signature of Sacubitril/Valsartan in healthy humans. The findings reveal a distinctive perturbation in pyrimidine metabolism, suggesting possible links to drug mechanisms relevant to cardiac cell cycle regulation. These results underscore the utility of untargeted pharmacometabolomics in uncovering systemic drug effects and highlight potential biomarkers for monitoring therapeutic response or guiding precision treatment strategies in heart failure.

## 1. Introduction

Sacubitril/Valsartan are widely used to improve clinical outcomes in heart failure patients. This drug combination significantly reduces mortality and hospitalization rates primarily by impacting the renin–angiotensin–aldosterone system (RAAS) and natriuretic peptide systems. Acting as both a neprilysin inhibitor (Sacubitril) and an angiotensin II receptor blocker (Valsartan), this dual mechanism provides potential synergistic benefits compared to targeting either pathway alone [1]. The RAAS orchestrates key physiological processes in cardiac, vascular, and renal function by regulating vascular tone and fluid balance [2], whereas natriuretic peptides oppose these effects and support vasodilation and natriuresis. ANRI represents a significant advancement in the management of chronic heart failure (HF), particularly over the past two decades. This angiotensin receptor–neprilysin inhibitor (ARNI) has demonstrated favorable effects on cardiac function in patients with concomitant renal impairment and HF. The United Kingdom Heart and Renal Protection-III (UK HARP-III) trial reported that Sacubitril/Valsartan produced renal outcomes comparable to those of irbesartan, including preservation of kidney function and reduction in proteinuria. Additionally, it resulted in further reductions in blood pressure and significantly improved cardiovascular biomarkers such as high-sensitivity troponin I and N-terminal pro-B-type natriuretic peptide (NT-proBNP), highlighting its potential to modulate both cardiac and renal risk profiles in patients with chronic kidney disease (CKD) and HF [3].

Despite well-documented clinical efficacy [2], knowledge of the drug’s metabolic effects remains limited. To address the limited understanding of the metabolic impact of Sacubitril/Valsartan in humans, recent studies have begun to explore its pharmacometabolomic profile in clinical populations. In a prospective metabolomics study of patients with end-stage renal disease (ESRD) and heart failure (HF), investigators evaluated serum samples collected before and after treatment with Sacubitril/Valsartan. Patients were stratified based on clinical response into good responders (GRs) and poor responders (PRs). Metabolomic profiling identified nine significantly altered metabolites between the GR and PR groups prior to treatment initiation, suggesting potential early biomarkers of drug responsiveness. Notably, three lysophosphatidylcholine (LysoPC) species demonstrated strong predictive performance based on machine learning algorithms, including random forest and support vector machine models. Furthermore, expression of the enzyme phospholipase A2 group IVA (PLA2G4A), linked to LysoPC metabolism, was significantly upregulated in the PR group. These findings imply that dysregulated lipid metabolism may impair therapeutic efficacy of Sacubitril/Valsartan and highlight PLA2G4A as a possible target for enhancing drug sensitivity in patients with ESRD and HF [4]. One study showed that Sacubitril/Valsartan influence glucose and lipid metabolism in heart failure patients both with and without diabetes mellitus, as well as the metabolism of natriuretic peptides [5]. This study is the first to characterize the acute pharmacometabolomic signature of Sacubitril/Valsartan in healthy human volunteers, providing foundational evidence that the drug modulates key biosynthetic and proliferative pathways. These findings could inform future research into long-term effects, drug repurposing, and individualized treatment strategies in heart failure management. Metabolomics is a comprehensive approach to analyzing small molecules produced by cellular processes and offers a powerful means to illuminate these effects [6,7,8,9,10].

Pharmacometabolomics, which applies metabolomics to drug response, has demonstrated utility in identifying biomarkers predictive of medication efficacy and in monitoring pharmacological effects [11,12]. Prior metabolomics studies have investigated the impact of various agents (e.g., metformin, dexamethasone) on healthy volunteers [13,14,15]. For Sacubitril/Valsartan, work in rat models of chronic heart failure has identified alterations in tryptophan metabolism and inflammation [16,17]. However, human data remain scarce.

Here, we used an LC-QToF-based untargeted metabolomics approach to elucidate the kinetic metabolic changes associated with Sacubitril/Valsartan in healthy volunteers. This metabolic profile will help in better understanding the pathways involved in this treatment to map them out with potential side effects or alternative therapeutic targets for drug repurposing.

## 2. Results

### 2.1. Clinical and Demographic Data of Study Subjects

Table 1 summarizes the clinical and demographic data for the 14 healthy male volunteers enrolled in this study twice with a two-week washout period. All biochemical test results were obtained once at the screening stage before they became involved in this study. Fasting blood sugar, urea, creatinine, sodium, potassium, aspartate transaminase (SGOT), serum glutamate pyruvate transaminase (SGPT), alkaline phosphatase (ALP), and total bilirubin were within normal limits. Participant ages ranged from 23 to 51 years, and 50% reported being smokers. None tested positive for ketones.

### 2.2. Metabolomic Analysis

**Figure 1:**
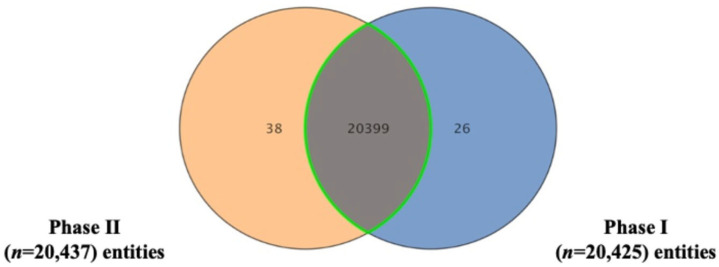
Venn diagram representing the count of shared and unique metabolic features between the two experimental phases, following data filtering. The overlap of mass ions (*n* = 20,399) detected in Phase I (*n* = 20,425) and Phase II (*n* = 20,437) under both positive and negative ionization modes, as analyzed by Mass Profiler Professional (MPP) software.

Metabolomics analysis was conducted in two phases involving the same 14 healthy volunteers, with a two-week washout period separating the phases. Across both phase samples, a total of 20,475 mass ion features were detected: 13,453 under positive ionization and 7022 under negative ionization. Of these, 20,399 features were commonly detected in both phases, as shown in the Venn diagram (Figure 1). Features with missing values based on frequency in more than 80% of the study groups were evaluated (*n* = 13,840) for further analysis.

**Figure 2:**
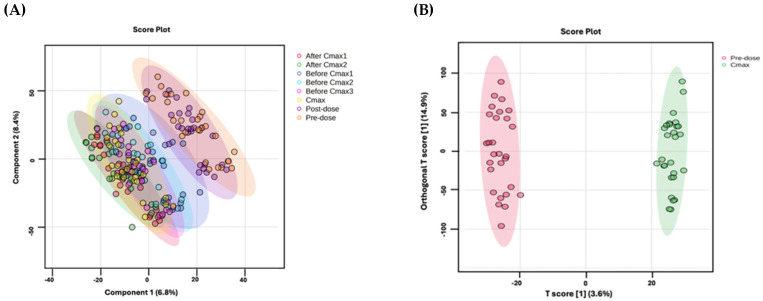
(**A**) PLS-DA plots of the metabolic profiles of 14 healthy subjects over two experiments, showing distinct clustering at eight time points following two doses of Sacubitril/Valsartan. (**B**) OPLS-DA compares pre-dose (red) vs. Cmax (green) profiles, revealing a clear separation (R^2^Y = 0.993, Q^2^ = 0.768). Model robustness was evaluated using a 100-permutation test.

A partial least-squares discriminant analysis (PLS-DA) was performed on 20,399 metabolic features from 14 healthy volunteers who received two doses of Sacubitril/Valsartan at eight time points: pre-dose, three intervals leading up to the maximum concentration (Cmax), the Cmax itself, two intervals post Cmax, and 48 h post dose. As shown in Figure 2A, the PLS-DA plot distinguishes these sampling points clearly. Notably, samples collected near Cmax form a cluster separate from those pre-dose and after 48 h, indicating distinct metabolic profiles that reflect changes in drug availability over time.

Orthogonal projections to latent structures discriminant analysis (OPLS-DA) was applied to compare pre-dose vs. Cmax metabolic profiles across all 14 subjects. As shown in Figure 2B, the model demonstrates a clear separation of these two time points (Q^2^ = 0.768, R^2^Y = 0.993), indicating a robust and highly reliable capability to distinguish between the metabolic states associated with drug administration.

**Figure 3:**
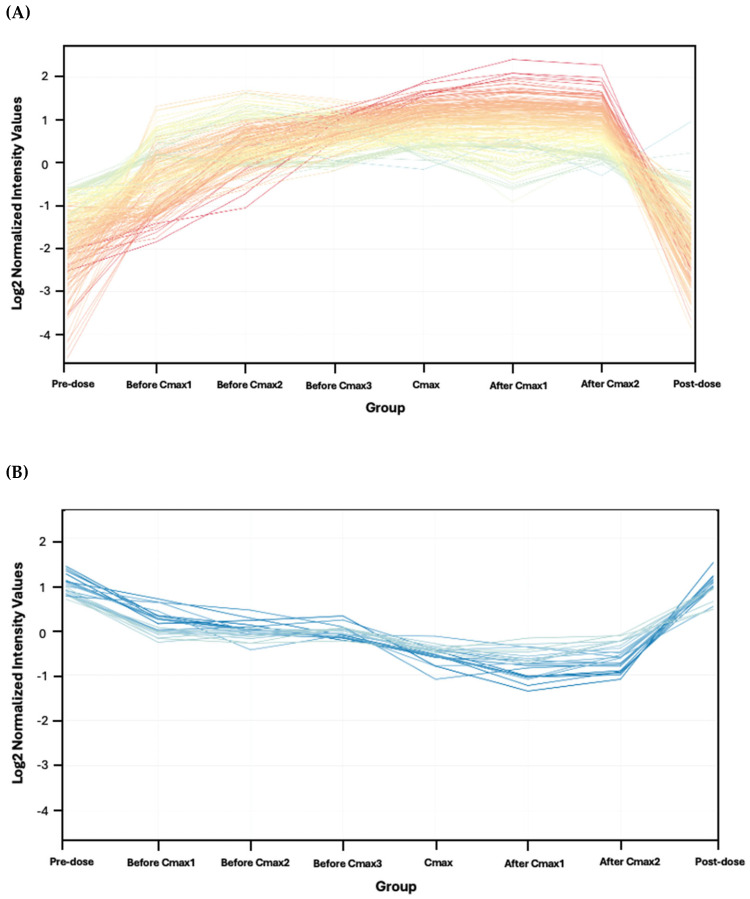
Metabolomics profiles of the 258 features meeting the ≥2 fold-change criterion between pre-dose and Cmax. (**A**) A total of 235 upregulated features. (**B**) A total of 23 downregulated features. The intensity of the color associated with the statistical significance, where the darker means the most significant.

**Figure 4:**
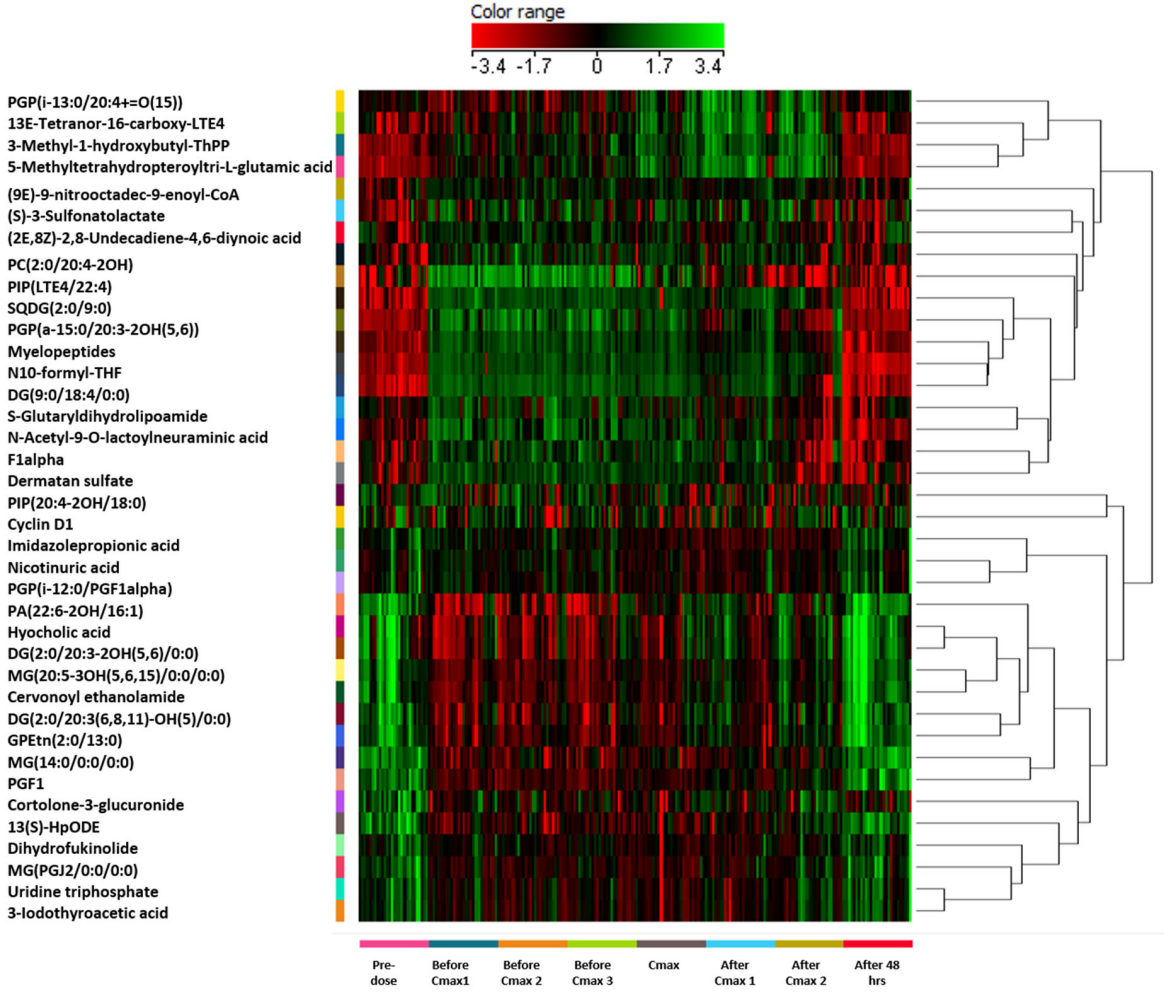
Hierarchical clustering and heatmap analysis of the 31 significantly dysregulated endogenous metabolites identified following Sacubitril/Valsartan administration in healthy subjects. Metabolite intensities were normalized and visualized across eight individualized time points: pre-dose, three time points before Cmax (before Cmax 1–3), at Cmax, two time points after Cmax (after Cmax 1–2), and 48 h post dose. Clustering was performed using Pearson correlation distance and average linkage to group both metabolites (rows) and time points (columns). Color-coding reflects the normalized expression level of each metabolite: red indicates upregulation (positive deviation from the median), green indicates downregulation (negative deviation from the median), and black represents expression levels close to the median. The color range spans from –3.4 (green, minimum relative abundance) to +3.4 (red, maximum relative abundance), as indicated by the color bar at the top of the figure. Key dysregulated metabolites include uridine triphosphate (UTP), associated with pyrimidine metabolism and cell cycle regulation. All pathway names and metabolite abbreviations are fully defined within the figure or accompanying Supplementary Table.

One-way ANOVA with Tukey’s post hoc analysis (FDR *p* < 0.05) was performed on 13,840 features across eight time points, identifying 315 significantly dysregulated features compared to background (Table S1). Fold-change analysis (≥2) of these 315 features between Cmax and pre-dose highlighted 258 features (235 upregulated, 23 downregulated; Figure 3 and Table S2). After excluded exogenous metabolites such as Sacubitril (drug), Valsartan (drug), and sacubitrilat (Sacubitril metabolite) Of these, 31 were successfully annotated as human endogenous metabolites (Figure 4 and Table S3), with 22 upregulated and 9 downregulated.

### 2.3. Pathway and Functional Analysis

**Figure 5:**
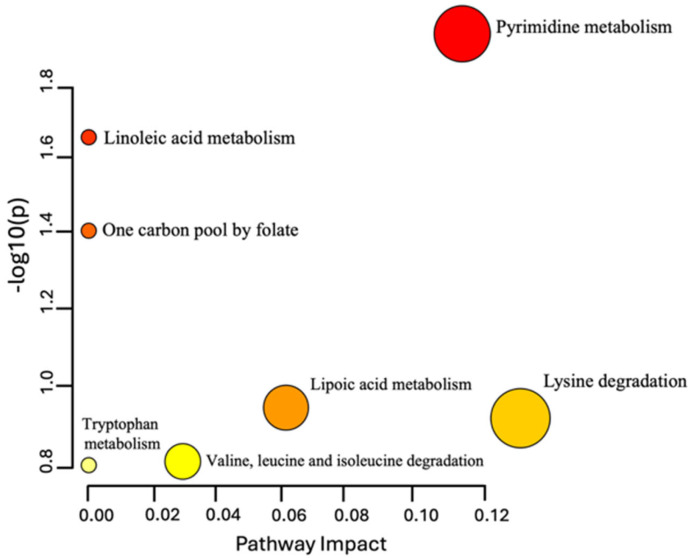
Pathway analysis of the 31 dysregulated endogenous metabolites following Sacubitril/Valsartan administration. Color intensity (yellow to red) reflects the significance level of each metabolite within the identified pathways.

Seven metabolic pathways were identified among the 31 Sacubitril/Valsartan-dependent metabolites (Figure 5 and Table S4). Notably, pyrimidine metabolism exhibited the most pronounced alterations, with uridine triphosphate (UTP) emerging as one of the most prominently dysregulated metabolites.

## 3. Discussion

Sacubitril combined with Valsartan is widely used formula to improve outcomes in heart failure patients, yet its effects on metabolic pathways remain insufficiently characterized. In this study, we aimed to identify dysregulated metabolites associated with the administration of this combination therapy, thereby providing insights into potential side effects and enabling more targeted treatment strategies. Employing an LC-QToF HRMS approach, we identified novel metabolic patterns that illuminate the biological mechanisms underlying Sacubitril/Valsartan’s clinical efficacy and potentially its adverse effects in heart failure. These insights could ultimately guide more refined patient management and personalized pharmacological interventions.

Previous investigations in heart failure patients, both with and without diabetes mellitus, revealed that Sacubitril/Valsartan disrupts glucose, lipid, and natriuretic peptide metabolism [5]. Nonetheless, the precise mechanisms that mediate these benefits are not fully understood. As shown in Figure 2B, multivariate modeling using OPLS-DA revealed a clear metabolic separation between pre-dose and Cmax samples, highlighting a distinct and reproducible drug-induced shift in the plasma metabolome. This indicates a robust pharmacometabolomic effect, even in healthy individuals. Our study identified 31 dysregulated endogenous metabolites—primarily amino acids, peptides, and lipids, with noteworthy alterations in pyrimidine metabolism (Figure 5). Figure 4 illustrates the clustering of these dysregulated metabolites, with visible temporal changes that align with individualized pharmacokinetic profiles. Heightened production, conversion, and transport of pyrimidine molecules suggest increased cellular growth demands, particularly relevant in the context of heart failure pathology.

Among the most prominently dysregulated metabolites was UTP, which plays a central role in pyrimidine metabolism as a precursor for other nucleotides vital for DNA and RNA synthesis [18]. Elevated UTP and other pyrimidine nucleotides can accelerate cellular proliferation, underscoring their potential importance in both physiological and pathological remodeling processes [18]. Consequently, targeting pyrimidine metabolism may provide therapeutic strategies to attenuate hypertrophy, limit fibrosis, and bolster cardiac function [18,19].

The dual mechanism of Sacubitril/Valsartan via neprilysin inhibition plus angiotensin II receptor blockade promotes cardiac repair by reducing cardiac strain through RAAS modulation and enhancing natriuretic peptides. These changes support pathways linked to cell cycle activation and cardiomyocyte regeneration [20]. In heart failure, compromised cardiomyocyte turnover exacerbates disease progression. By driving cell cycle re-entry and regulating proteins essential for cell division, Sacubitril/Valsartan may mitigate myocardial necrosis, potentially improving tissue viability and clinical outcomes. Indeed, reactivating the cardiomyocyte cell cycle can promote functional recovery after cardiac injury [20]. This targeted shift could reduce necrosis and reverse some of the maladaptive remodeling commonly seen in advanced heart failure.

Our findings underscore the pivotal role of pyrimidine metabolism in heart failure and suggest that Sacubitril/Valsartan can modulate this pathway, thereby supporting cardiac repair. Figure 2B and Figure 4 collectively provide visual evidence of the drug’s temporal metabolic impact and its influence on pyrimidine and proliferative signaling pathways. This study offers several distinctive strengths that advance current pharmacometabolomics research. First, it represents the first-in-human untargeted metabolomics investigation of Sacubitril/Valsartan administration in healthy volunteers, providing foundational insight into the acute systemic metabolic responses to this widely prescribed heart failure therapy. Second, the study design employed individualized pharmacokinetic profiling to guide sampling time points, allowing for high-resolution temporal mapping of drug-induced metabolic changes. Furthermore, this subject-specific approach, focusing on personalized Tmax and Cmax intervals enabled detection of subtle, yet biologically meaningful, metabolite fluctuations that would likely be obscured using conventional fixed time points. Third, the integration of both multivariate and univariate statistical models, including OPLS-DA with permutation validation and FDR-corrected ANOVA, strengthens the analytical robustness and confidence in the observed metabolic signatures. The clear separation of pre-dose and Cmax metabolic states (Figure 2B), alongside well-clustered dysregulated metabolites (Figure 4), highlights the reliability of the results. Fourth, this study employed high-resolution LC–QToF HRMS technology with stringent metabolite annotation criteria based on multiple public databases, enhancing the credibility of identified features. Lastly, the focus on pyrimidine metabolism and cell cycle regulators such as UTP fills a critical knowledge gap in understanding the proliferative and biosynthetic processes potentially modulated by Sacubitril/Valsartan. These findings open new avenues for mechanistic investigation and therapeutic repurposing. Together, these strengths make the current study a valuable reference for future research on cardioactive pharmacotherapies and exemplify how precision medicine approaches can be applied even in early-phase, healthy volunteer settings.

While the design enabled the detection of temporal metabolic alterations associated with drug exposure, several limitations should be acknowledged. First, the sample size was small (*n* = 14) and limited to male subjects, which restricts the generalizability of the findings across sexes. Metabolic responses can differ between males and females due to hormonal and physiological variations, and future studies should incorporate sex-balanced cohorts to assess potential sex-specific drug responses. Second, 50% of participants were self-reported smokers, and while no statistically significant differences in pyrimidine metabolism were observed between smokers and non-smokers within this sample, the small subgroup size precluded rigorous stratified analysis. Smoking is known to affect several metabolic pathways, including nucleotide metabolism; therefore, the influence of smoking status warrants further investigation in larger, stratified studies. Third, although this study employed a two-period crossover design to enhance reproducibility and minimize inter-individual variability, it is important to recognize potential confounding effects from diurnal variation in metabolite levels. All dosing and sample collections were conducted in the early morning in the fasted state, and meals were standardized, minimizing time-of-day and fed-state variability. However, subtle circadian influences on metabolism cannot be fully excluded without continuous sampling over a 24 h period. Fourth, only a single dose of Sacubitril/Valsartan was administered, and thus this study reflects acute, short-term pharmacometabolomic effects in healthy individuals. The relevance of these findings to chronic dosing or patient populations with heart failure remains uncertain. While the observed upregulation of pyrimidine metabolism and dysregulation of cell cycle markers (e.g., UTP) suggest possible links to cardiac remodeling, this hypothesis requires further validation in clinical settings.

Our finding show PIP (20:4-2OH/18:0) is a derivative of phosphatidylinositol phosphate lipids, which are essential for intracellular signaling and membrane trafficking. Alterations in PIP metabolism have been linked to cardioprotective mechanisms, particularly via PI3K/Akt signaling, which is upregulated in response to natriuretic peptide activation and can mediate cardiomyocyte survival and anti-hypertrophic effects [21,22].

Ether-linked and hydroxylated lipid species, such as PGP(a-15:0/20:3-2OH) and DG(2:0/20:3-2OH/0:0), though infrequently reported, may indicate modulation of redox-sensitive lipid pathways, possibly reflecting the attenuation of oxidative stress seen with ARNi therapy. Sacubitril/Valsartan has been shown to reduce lipid peroxidation and improve mitochondrial efficiency in both clinical and experimental models [23].

These changes may also involve lipid mediator resolution pathways, as DG and PGP species can act as intermediates in the biosynthesis of eicosanoids, resolvins, and other inflammation-resolving lipids. This is consistent with reports that ARNi reduces systemic and cardiac inflammatory cytokine expression [24].

Finally, although advanced multivariate and univariate analyses were used to identify significant metabolic changes, subtle inter-individual variability in metabolite expression could still influence the clustering and model performance. Moreover, the annotations of metabolites were based on high-resolution MS data and public databases without MS/MS confirmation for all features, which may introduce identification uncertainties in some cases. In conclusion, while this pilot study demonstrates the utility of untargeted metabolomics to explore drug-induced metabolic shifts, it also underscores the need for larger, sex-inclusive, and clinically diverse cohorts to validate these findings and explore their translational significance.

## 4. Materials and Methods

### 4.1. Subject Recruitment and Study Design

Healthy volunteers were recruited following clinical laboratory testing and comprehensive screening protocols in accordance with ICH-GCP standards. Eligible participants were non-smoking males and females aged 18 to 45 years, with body mass index (BMI) between 18.5 and 27.0 kg/m^2^ and no clinically significant abnormalities upon physical examination, electrocardiogram, or routine hematological and biochemical parameters.

All volunteers enrolled in this study were required to be in good general health, with no history of acute or chronic illnesses, and with normal hepatic and renal function confirmed by clinical laboratory testing. Participants were excluded if they had taken any prescription or over-the-counter medications within two weeks prior to dosing. Female subjects of childbearing potential were eligible only if they tested negative for pregnancy at screening. Additional exclusion criteria included a known hypersensitivity to Sacubitril, Valsartan, or related agents; participation in another clinical trial within the previous 60 days; recent blood donation exceeding 500 mL within 8 weeks; a history of alcohol or substance abuse; smoking within six months; and any gastrointestinal, hepatic, renal, or cardiovascular disorders that could potentially influence drug metabolism or absorption. Volunteers were also excluded if screening results revealed abnormal fasting glucose levels; seropositivity for hepatitis B, hepatitis C, or HIV; or if they had a known intolerance to components of the standardized meals. Comprehensive screening was conducted within 21 days prior to dosing and included a detailed medical history, physical examination, electrocardiogram, vital signs assessment, and laboratory evaluations of hematology, biochemistry, urinalysis, and infectious disease markers.

**Scheme 1:**
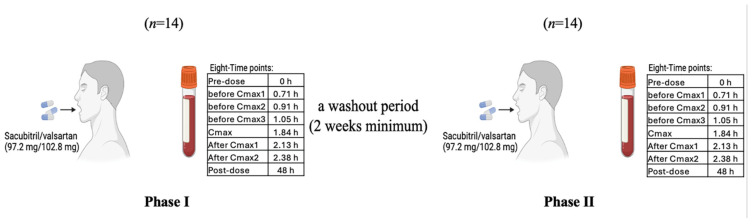
Study of Sacubitril/Valsartan (97.2 mg/102.8 mg) in healthy volunteers (*n* = 14), with eight sampling points per phase and a ≥ 2-week washout period.

Fourteen healthy volunteers passed the extensive health screening and were recruited for this study. In Phase I (“Experiment 1”), each received a single 200 mg film-coated tablet containing 97.2 mg Sacubitril and 102.8 mg Valsartan (Novartis Farma, Italy) (Scheme 1). After a two-week washout period, the same volunteers repeated the same experiment with an identical dose in Phase II (“Experiment 2”). This study was conducted twice to enhance the statistical robustness and reproducibility of the findings, which is especially important when evaluating the pharmacometabolomic impact of a combination drug such as Sacubitril/Valsartan. Blood samples were kept at room temperature (approximately 20–22 °C) for no longer than 30 min prior to centrifugation for plasma separation. Following centrifugation, plasma samples were aliquoted and immediately stored at −80 °C. Samples remained frozen for a maximum of six months before analysis to preserve integrity. Plasma was collected at multiple time points; however, only eight time points, selected based on the individual mean Tmax of both drugs, were included in this study: pre-dose, before Cmax1, before Cmax2, before Cmax3, at Cmax, after Cmax1, after Cmax2, and 48 h post dose.

### 4.2. Chemical and Material

LC-MS-grade acetonitrile (ACN), methanol (MeOH), formic acid, sodium formate (HCOONa), LockSpray [Leucine-enkephalin], and distilled water were purchased from Fisher Scientific (Ottawa, ON, Canada). Uridine triphosphate (UTP) and cholic acid standard materials were purchased from Sigma (St. Louis, MO, USA).

### 4.3. Sample Preparation

**Scheme 2:**
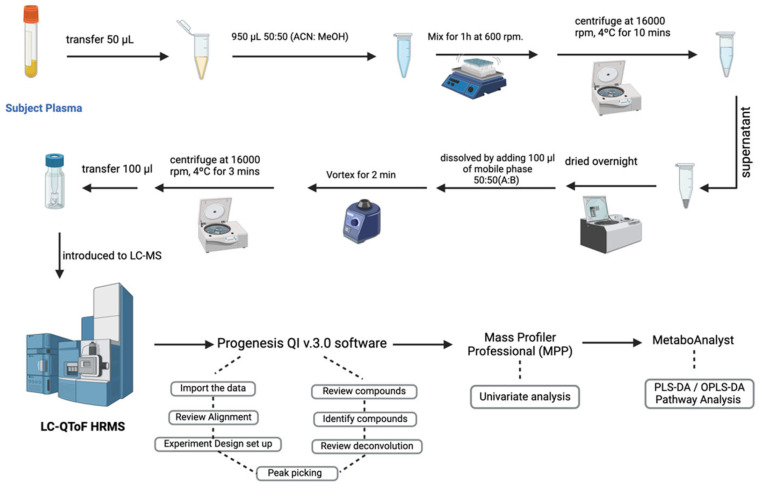
Overview of the study’s workflow for sample collection, processing, LC-HRMS analysis, and data interpretation.

Metabolites from 224 volunteer and 20 pooled quality control (QC) samples were extracted following the lab’s standard protocol and stored at −80 °C [25]. In detail, 50 µL of plasma was transferred into an Eppendorf tube. The metabolites were extracted by adding 950 µL of the extraction solvent 1:1 *v/v* (ACN: MeOH). Afterward, the samples were placed on the shaker for 1 hr at 25 °C in a Thermomixer at 600 rpm (Eppendorf, Germany). The slurry was centrifuged at 16,000 r.p.m and 4 °C for 10 min, and the supernatant was transferred into a new Eppendorf tube to be evaporated using a speed vacuum overnight (SpeedVac; Christ, Germany). The dried extract, stored at −80 °C, was reconstituted for metabolomics analysis with 100 µLof mobile phase (1:1) A:B [A: 0.1% formic acid in dH_2_O—B: 0.1% formic acid in 1:1 *v/v* MeOH and ACN] (Scheme 2).

### 4.4. Liquid Chromatography with Quadrupole Time-of-Flight Mass Spectrometry (LC-QToF HRMS)

The sample’s metabolome was profiled using a Waters Acquity ultra-performance liquid chromatography (UPLC) system coupled with a Xevo G2-S QTOF mass spectrometer equipped with an electrospray ionization source (ESI). A 5 μL sample was injected into an ACQUITY UPLC HSS T3 (100 × 2.1 mm 1.8 μm) column (Waters Ltd., Elstree, UK). The mobile phase was composed of 0.1% formic acid in dH_2_O as solvent A, and B consisted of 0.1% formic acid in 1:1 *v/v* ACN: MeOH. A gradient elution schedule was run as follows: 0–16 min 95- 5% A, 16–19 min 5% A, 19–20 min 5–95% A, and 20–22 min 5- 95% A, at 300 µL/min flow rate. MS spectra were acquired under positive and negative electrospray ionization modes (ESI+, ESI−). MS conditions were as follows: source temperature was 150 °C, the desolvation temperature was 500 °C for both ESI, the capillary voltage was 3.20 kV (ESI+) or 3 kV (ESI−), cone voltage was 40 V, desolvation gas flow was 800.0 L/h, and cone gas flow was 50 L/h. The collision energies of low and high functions were set at off and 10 V to 50 V, respectively, in MS^E^ mode. The mass spectrometer was calibrated with sodium formate in 100–1200 Da. Data were collected in continuum mode with a Masslynx™ V4.1 workstation (Waters Inc., Milford, MA, USA). QCs were introduced to the instrument randomly to validate the system’s stability [18]. After that, they were analyzed following the routine protocol. The acceptance criteria were to have all the QC samples separated from the other study groups, clustered together, and use their relative standard deviations (RSD%) < 40% [25].

### 4.5. Metabolite Identification and Enrichment Pathway Analysis

The raw mass spectrometry data were processed using Progenesis QI v3.0 (Waters Technologies, Milford, MA, USA). Initial steps included alignment of ion signals based on retention time and mass-to-charge ratio (m/z), followed by automated peak picking and deconvolution using default parameters to ensure high-quality feature detection. To improve data reliability, only features detected in ≥80% of the samples were retained, and those with low abundance or poor signal quality were excluded using Mass Profiler Professional (MPP) software v15.0 (Agilent Inc., Santa Clara, CA, USA).

Metabolite annotation was performed using Progenesis QI (Waters Corporation, Milford, MA, USA), which integrates accurate mass measurement, isotopic pattern recognition, adduct detection, and MS/MS fragmentation analysis. Identification was based on the comparison of precursor and fragment ion masses, isotopic distributions, and fragmentation patterns against established public databases, including the Human Metabolome Database (HMDB), METLIN MS/MS (accessed 14 December 2023), LipidMaps, LipidBlast, and KEGG. A mass error threshold of ±5 ppm was applied. Annotation confidence was further enhanced using both empirical and in silico fragmentation tools embedded in the software, with progenesis fragmentation scores and isotope similarity contributing to overall annotation ranking.

All metabolite identifications were assigned as putative (MSI Level 2), in accordance with the Metabolomics Standards Initiative [26], as authentic standards were not analyzed under matched chromatographic and instrumental conditions. No MSI Level 1 identifications were reported.

To complement annotation-dependent pathway analysis, a complementary enrichment analysis was conducted using the mummichog algorithm (version 2.0) implemented in MetaboAnalyst 5.0. This method enables functional interpretation of untargeted metabolomics data by linking statistically significant m/z features directly to biological pathways without requiring prior metabolite identification. Significant features (FDR < 0.05) were mapped against the Homo sapiens KEGG pathway library, and pathway significance was determined based on overrepresentation analysis and activity scoring.

### 4.6. Statistical Analysis

To account for inter-individual variability in drug metabolism, sampling time points were selected relative to each participant’s personalized pharmacokinetic profile, with a particular focus on individualized Tmax and Cmax. This subject-specific timing allowed for a more accurate kinetic mapping of metabolite fluctuations during the early, peak, and late post-dose phases.

Raw metabolite abundance data were processed and analyzed using MetaboAnalyst 6.0 (McGill University, Montreal, Canada; http://www.metaboanalyst.ca) (accessed 13 December 2023). Prior to statistical modeling, data were median-normalized, log-transformed, and Pareto-scaled to approximate a normal distribution and minimize the influence of high-abundance features.

Multivariate statistical analyses were performed to explore overall metabolic variation across time points. Both partial least-squares discriminant analysis (PLS-DA) and orthogonal projections to latent structures discriminant analysis (OPLS-DA) models were constructed to evaluate temporal clustering and separation patterns. Model performance was assessed using 100-permutation tests, and the key parameters-R^2^Y (explained variance) and Q^2^ (predictive ability) were reported to assess model robustness and generalizability.

Complementary univariate analysis was performed using Mass Profiler Professional v15.0 (Agilent Technologies, Santa Clara, CA, USA). Time-dependent differences in metabolite levels were assessed using one-way ANOVA, followed by Tukey’s post hoc test. Statistical significance was determined using false discovery rate (FDR) correction, with a threshold of FDR-adjusted *p* < 0.05. Additionally, features exhibiting a fold change ≥ 2 between pre-dose and Cmax were prioritized for further biological interpretation. To visualize dynamic changes, hierarchical clustering and heatmap analysis were conducted based on Pearson correlation distance and average linkage method. Pathway analysis was then applied to the subset of significantly dysregulated endogenous metabolites to identify the most affected metabolic pathways following Sacubitril/Valsartan administration.

## 5. Conclusions

This study is the first to employ an MS-based untargeted metabolomics approach to characterize the dynamic metabolic effects of Sacubitril/Valsartan in healthy volunteers. Our findings highlight the upregulation of the pyrimidine metabolism pathway, with UTP emerging as potential cell cycle-related metabolic signature influenced by this therapy. While Sacubitril/Valsartan has demonstrated an ability to counteract certain metabolic dysregulations observed in heart failure, further investigation is needed to clarify its direct impact on pyrimidine metabolism and to identify additional modulated metabolites. Advanced metabolomics techniques will be pivotal in refining our understanding of Sacubitril/Valsartan’s mechanisms of action and uncovering new therapeutic targets to improve heart failure management.

## Figures and Tables

**Table 1:** Clinical and demographic characteristics of the 14 recruited subjects.

Clinical and Demographic Data	Mean (*n =* 14)	Standard Deviation	Normal Range *
Age (y)	33.64	7.13	NA
Height (m)	1.70	0.07	NA
Weight (Kg)	77.86	10.00	NA
Smoker (%)	50%	-	0%
BMI (Weight/Height^2^)	26.76	2.23	NA
Fasting Blood Sugar (mg/dL)	95.21	7.83	70.00–115.00
Urea (mg/dL)	30.66	5.69	10.0–50.0
Creatinine (mg/dL)	1.13	0.08	0.60 –1.30
Sodium (mmol/L)	141.07	1.53	135–153
Potassium(mmol/L)	4.27	0.45	3.50–5.30
Aspartate transaminase (SGOT) (U/L)	23.43	5.22	Up to 42
Serum Glutamate Pyruvate Transaminase (SGPT) (U/L)	26.64	5.23	Up to 50
Alkaline phosphatase (ALP) (IU/L)	103.64	26.30	40–150
Bilirubin Total (mg/dL)	0.44	0.12	Up to 1.40

* According to Jordan Center for Pharmaceutical Research (JCPR) and the Jordanian FDA regulations and rules.

## Data Availability

The data presented in this study are available on request from the corresponding author. The data are not publicly available due to privacy and ethical restrictions related to human sample handling and confidentiality agreements.

## Supplementary Materials

The following supporting information can be downloaded at: https://www.mdpi.com/article/10.3390/ph18091264/s1, The following supporting information can be found online Table S1: Overview of One Way ANOVA results for Sacubitril combined with Valsartan. Contains data on compounds, retention times, masses, *p*-values, fold changes, and regulation; Table S2: Fold change between Cmax vs. Pre-dose; Table S3: Endogenous metabolites; Table S4: Pathway and Functional Analysis.
